# *Fusarium graminearum*^1^H NMR metabolomics

**DOI:** 10.1016/j.dib.2018.04.112

**Published:** 2018-05-01

**Authors:** A. Sevastos, I.F. Kalampokis, A. Panagiotopoulou, M. Pelecanou, K.A. Aliferis

**Affiliations:** a Laboratory of Pesticide Science, Department of Crop Science, Agricultural University of Athens, Athens, Greece; b Institute of Biosciences & Applications, National Centre for Scientific Research “Demokritos”, Athens, Greece; c Department of Plant Science, Macdonald Campus of McGill University, Sainte-Anne-de-Bellevue, Quebec, Canada

## Abstract

Raw ^1^H NMR spectra of *Fusarium graminearum* hyphae can be found at the website of the pesticide metabolomics group (PMG) of the Agricultural University of Athens at the address: http://www.aua.gr/pesticide-metabolomicsgroup/Resources/Fusarium_graminearum_NMR_spectra.html, accession number PMG-01–17. The data set support the research article “Implication of *Fusarium graminearum* Primary Metabolism in its Resistance to Benzimidazole Fungicides as revealed by ^1^H NMR Metabolomics” [1].

**Specifications table**

**Table:** 

Subject area	*Biology*
More specific subject area	*Fungal metabolomics*
Type of data	^1^H NMR spectra in *.jdx format
How data was acquired	^1^H NMR spectroscopy, Bruker Avance spectrometer at 500 MHz
Data format	Raw
Experimental factors	*Fusarium graminearum* wild and carbendazim-resistant strains following or not exposure to the fungicide
Experimental features	Hyphae of a *Fusarium graminearum* wild and three carbendazim-resistant strains were treated with different concentrations of the fungicide. In total three pooled samples (obtained from fifteen biological replications), and a quality control (QC) sample were obtained for each of the treatments.
Data source location	Athens, Greece
Data accessibility	The data are available at the website of the pesticide metabolomics group (PMG) of the Agricultural University of Athens at the address: http://www.aua.gr/pesticide-metabolomicsgroup/Resources/Fusarium_graminearum_NMR_spectra.html, accession number PMG-01–17.
Related research article	A. Sevastos^1^, I. Kalampokis^1^, A. Panagiotopoulou^2^, M. Pelecanou^2^, K. A. Aliferis^3‡*^, 2017. Implication of *Fusarium graminearum* Primary Metabolism in its Resistance to Benzimidazole Fungicides as revealed by ^1^H NMR Metabolomics. Pesticide Biochemistry and Physiology (*In Press*)

**Value of the data**

• Data could be used for further experiments towards understanding fungal metabolism in relation to fungicide resistance.
• Data could be used to compare responses of different fungal species to pesticide toxicity.
• Data could be combined with analytical data obtained by other analyzers in order to obtain a global overview of fungal metabolism.

## 1 Data

The data set is composed of raw ^1^H NMR spectra of one *Fusarium graminearum* wild and three carbendazim-resistant strains which were treated or not with sub-lethal doses of the fungicide (2 mg L^−1^) and 10 mg L^−1^. In total three pooled samples (obtained from fifteen biological replications), and a quality control (QC) sample were obtained for each of the treatments.

## 2 Experimental design, materials, and methods

### 2.1 Chemicals, reagents and inhibitors

Deuterium oxide (D_2_O) of 99.9% purity, containing 0.05% v/v 3-(trimethylsilyl)-propionic acid-2,2,3,3-d4-sodium salt (TSP) (Sigma-Aldrich, St. Gallen, Switzerland), was used in ^1^H NMR analyses. The fungicide carbendazim (99.0%, v/v) was kindly provided by Bayer CropScience AG (Monheim am Rhein, Germany). Stock solutions of carbendazim were prepared in HPLC-grade ethanol (Thermo Fisher Scientific GmbH, Darmstadt, Germany) at a concentration of 300 μg mL^−1^, which were stored at −25 °C until further use.

### 2.2 Biological material

The isolate CBS 110261 of *F. graminearum* (wild type, *WT*) was obtained from the CBS Fungal Biodiversity Centre (Royal Netherlands Academy of Arts and Sciences, KNAW, Amsterdam, The Netherlands). The *F. graminearum* isolates *FG1*, *FG2*, *FG3*, and *FG6* had been obtained from the parental isolate CBS 110261 by UV mutagenesis as previously described [2].

### 2.3 Culture conditions and bioassays

Starter cultures of the *F. gramineraum* strains were grown on potato dextrose agar (PDA, Neogen, Auchincruive, Scotland, UK) in Petri plates (9 cm in diameter), at 25 °C in the dark. The inoculum that was used in the experiments was consisted of 5-mm in diameter culture plugs taken from the edges of 6-day old starter cultures using a cork borer. All handling and bioassays were performed under aseptic conditions in a laminar flow cabinet following good laboratory practice (GLP) and standard operating procedures (SOP). The plugs were then placed in the center of sterile cellophane membranes (9-cm in diameter, 500 PUT; UCB, North Augusta, USA) in a Petri plate (9-cm in diameter) containing 25 mL of PDA amended or not with 2 mg L^−1^, which is the *WT׳*s sub-lethal concentration [1], or 10 mg L^−1^ of carbendazim. The concentration of 10 mg L^−1^ carbendazim was only applied to *FG1* and *FG2*. In total, fifteen cultures were used per treatment (biological replications). Ten days after treatments, mycelia were carefully collected from the surface of the cellophane membranes using a spatula. Collected hyphae from five cultures were pooled to provide one pooled sample in plastic falcon tubes (50 mL), which was immediately flash frozen in liquid N_2_ for metabolism quenching. In total three pooled samples (obtained from the fifteen biological replications), and a quality control (QC) sample were obtained for each of the treatments. Samples were immediately extracted for ^1^H NMR metabolomics.

### 2.4 Sample preparation and extraction for ^1^H NMR metabolomics

Sample preparation and extraction for ^1^H NMR metabolomics was performed as previously described [3], with minor modifications. Briefly, the obtained pooled samples were pulverized to a fine powder in a mortar with a pestle using liquid N_2_ and transferred to falcon tubes (50 mL) and then, they were stored at −80 °C until further processing. For the removal of water, sub-samples (100 mg each) were lyophilized for 24 h. Polar metabolites were extracted by adding D_2_O (0.8 mL) to the lyophilized samples in Eppendorf tubes (1.5 mL). Initially, sonication was performed in an ultrasonic bath (Branson 1210, Branson Ultrasonics, Danbury, CT, USA) for 25 min. Then, extraction was continued for 2 h under agitation (120 rpm) at 24 °C. Debris were removed by centrifugation (12,000×*g* for 60 min at 4 °C) and the supernatants were collected and further purified by an additional centrifugation (12,000×*g* for 30 min at 4 °C). The extracts were kept in Eppendorf tubes (1.5 mL) at −80 °C until the ^1^H NMR spectra acquisition.

### 2.5 ^1^H NMR analyses

After thawing, extracts were added into NMR tubes (5 mm Thin Wall Precision NMR Sample Tubes 8" L, Wilmad, Vineland, NJ, USA) and immediately analyzed. ^1^H NMR spectra acquisition was performed using a Bruker Avance spectrometer at 500 MHz, equipped with a 5 mm inverse detection probe. The metabolic profiles were exemplified by ^1^H NMR spectra of aqueous extracts at 298 K. A total of 128 transients of 64 K data points were acquired per sample with a 90° pulse angle, a 2 s acquisition time and 2 s recycle delay, with presaturation of H_2_O during the recycle delay.

### 2.6 Spectra pre-processing, statistical analyses and biomarker discovery

^1^H NMR spectra pre-processing, multivariate analyses of data and biomarker discovery were performed as previously described [3], [4] with a few modifications. The obtained spectra were imported into the software Spectrus (Advanced Chemistry Development, Inc., ACD/Labs, Toronto, Canada), they were Fourier-transformed, and their phase and baseline were automatically corrected. Offsets of chemical shifts were corrected based on the reference signal of TSP (0.00 ppm). The pre-processed spectra were re-imported into the software for batch processing. The region between 0.91 and 8.36 *ppm* was integrated after the removal of regions such as, the one that corresponds to the water signal (4.70–4.80 *ppm*), and others for which no signals were recorded, in order to improve the quality and robustness of the analysis. For the retained regions, integration was performed by applying the Intelligent Bucketing algorithm of the software using the settings, width looseness; 50%, method; sum, width of bucket; 0.02 *ppm*. The identification of metabolites was based on chemical shifts, coupling constants (*J*) and also comparisons to ^1^H NMR spectra of analytical standards in D_2_O that had been analyzed in the same system under the same analytical conditions.

The obtained matrix was imported into the software SIMCA-P 13.0 software (Umetrics, MKS Instruments Inc., Andover, MA, USA) for multivariate data analyses and the discovery of trends and biomarkers based on partial least squares-discriminant analysis (PLS-DA). Prior to analyses, data were pareto scaled (1/HSD), which is the preferable normalization for NMR data. Cross validation was performed based on the default software settings and the corresponding values of the fraction of the sum of squares of *X׳s* and Y׳s explained by the current component (*R*^*2*^*X* and *R*^*2*^*Y*), and the predictive ability, *Q*_*(cum)*_^*2*^. The discovery of biomarkers was based on scaled PLS regression coefficients and standard errors were calculated by jack-knifing with 95% confidence interval [5]. The performance of the models was evaluated by the cumulative fraction of the total variation of the *X׳s* that could be predicted by the extracted components, Q_(cum)_^2^, and the *R*^*2*^*X* and *R*^*2*^*Y*.

In addition to PLS-DA, for the robust overview of the recorded metabolite profiles and their differences among treatments a cluster heat map in combination with two-dimensional (2D) hierarchical cluster analysis (HCA) was constructed using scripts of the Bioinformatics Toolbox of the software Matlab v.2016a (MathWorks, Natick, MA, USA). For HCA the linkage method of Ward was applied.

### 2.7 Metabolic network construction and metabolism monitoring

For the biological interpretation of the obtained information, a previously described approach was followed [6]. Since each metabolite could participate in more than one biosynthetic pathways, in order to gain an overview of its function within the global metabolism of a given biological system, such participation should be taken into consideration. Here, the participation of a metabolite in a biosynthetic pathway is described with the term “instance”, and for standardization purposes, the coding system and classification of the Kyoto Encyclopedia of Genes and Genomes (KEGG) for biosynthetic pathways (http://www.kegg.jp/kegg/pathway.html) was adapted.
