# Supplementary material for: Fusarium graminearum1H NMR metabolomics
Source: Data Brief. 2018 May 1;19:1162–5. doi: 10.1016/j.dib.2018.04.112 (PMC6139369; doi:10.1016/j.dib.2018.04.112)
Supplement: Supplementary file 1 — Supplementary material [file mmc1.docx]

Conflict of Interest

All authors confirm no conflict of interest.
